# The association between olfactory dysfunction and cardiovascular disease and its risk factors in middle-aged and older adults

**DOI:** 10.1038/s41598-020-80943-5

**Published:** 2021-01-13

**Authors:** Daeyoung Roh, Dong-Hee Lee, Soo Whan Kim, Sung Won Kim, Byung-Guk Kim, Do Hyun Kim, Ji-Hyeon Shin

**Affiliations:** 1 Mind-Neuromodulation Laboratory and Department of Psychiatry, Chuncheon Sacred Heart Hospital, Hallym University College of Medicine, Gangwon-do, Chuncheon-si, Republic of Korea; 2 Department of Otolaryngology-Head and Neck Surgery, College of Medicine, The Catholic University of Korea, Seoul, Republic of Korea

**Keywords:** Cardiology, Diseases, Medical research, Risk factors

## Abstract

While previous studies have reported olfactory dysfunction (OD) in relation to cardiovascular disease (CVD), few population-based studies have investigated whether such associations differ by sex. The purpose of this study was to identify the association between CVD and its risk factors with OD, and the sex-associated differences within the general population. We examined 20,016 adults aged 40 and older from the Korean National Health and Nutrition Examination Survey. All subjects reported on their history of OD. CVD and its risk factors included coronary artery disease (CAD), stroke, hypertension, diabetes, obesity, abdominal obesity, and hypertriglyceridemia; logistic regression was used to analyse their associations with OD, and additive interaction was used to analyse the interaction between risk factors and sex. In males, CAD was more likely to be associated with OD (odds ratio [OR] 1.81, 95% confidence interval [CI] 1.05–3.14), whereas abdominal obesity was associated with OD in females (OR 1.39, 95% CI 1.06–1.84).Additive interaction were observed between abdominal obesity and female sex with a relative excess risk of interaction of 0.45 (95% CI 0.26–0.63). Our findings suggest the relationship between OD and CVD and its risk factors and sex-associated differences among middle-aged and older adults.

## Introduction

Olfactory dysfunction is a common condition, affecting approximately 20% of the general adult population^1,2^. Olfaction decreases by degrees with aging, and is related to a reduced quality of life and critical health outcomes^2,3^. In older adults, poor olfaction is associated with higher long-term mortality, neurodegenerative diseases, and poor health outcomes^2,4^. In rhinologic fields, sino-nasal diseases are considered a major cause of olfactory dysfunction^2,5^. Other factors include head trauma, neurodegenerative diseases, toxins and medications, iatrogenic injuries, congenital disorders and normal aging^2,4,6^.

Accumulating evidence has suggested that cardiovascular health may be associated with olfactory dysfunction^3,7^. In older Americans, poor olfaction was reported to be associated with higher mortality from cardiovascular disease (CVD)^7^; however, some studies have reported that there was no association between CVD and olfactory dysfunction^8,9^. In a nationwide representative sample of American adults aged 40 years and older, CVD was not found to be associated with olfactory dysfunction^8^. Additionally, the reported associations between olfactory dysfunction and specific cardiovascular conditions have not been fully elucidated; thus, there were inconsistencies in these results. Therefore, to provide nationally presentative prevalence and association of olfactory dysfunction and CVD is needed. Further, little is known about the sex-specific association between olfactory dysfunction and CVD, despite the prevalence of gender-based differences regarding the conditions^1,8,10^.

The purpose of this population-based study of middle-aged and older Koreans was therefore to evaluate the prevalence and association between olfactory dysfunction and CVD and its risk factors and to assess whether these associations differ by sex.

## Results

### Socio-demographic and clinical characteristics

The socio-demographic characteristics of the study population (unweighted number of participants: 8587 males and 11,429 females) are listed in Supplementary Table S1; the presence of CVD and its risk factors are listed in Table 1. Over half of the population (57.1%) were female, with a mean age of 55.8 years. The female group was significantly older, with lower household incomes, lower education levels, and lower percentages of current smokers and heavy drinkers. Additionally, the female group reported a shorter sleep duration, and lower prevalence of rhinosinusitis were found to be less prevalent when compared to the male group; however, subjects expressing a lack of exercise were more frequently observed within the female group. For CVD and its risk factors, females had lower prevalence of diabetes, hypertension, CAD, stroke, obesity, hypertriglyceridemia and higher prevalence of abdominal obesity and low LDL levels compared with males.

**Table 1:** Presence of history of cardiovascular disease and its risk factors among Korean adults aged 40 and over, overall and stratified by sex.

Cardiovascular disease and its risk factors	Weighted prevalence % (95% CI)			P value for comparison between sex groups
	Total (n = 20,016)	Male (n = 8587)	Female (n = 11,429)	
**Diabetes**				< 0.001
Yes	10.4 (9.9–10.9)	11.2 (10.4–12.0)	9.6 (9.0–10.3)	
No	89.6 (89.1–90.1)	88.8 (88.0–89.6)	90.4 (89.7–91.0)	
**Hypertension**				< 0.001
Yes	35.5 (34.4–36.7)	36.4 (34.7–38.1)	34.7 (33.2–36.3)	
No	63.6 (63.3–65.6)	63.6 (61.9–65.3)	65.3 (63.7–66.8)	
**Coronary artery diseases**				0.037
Yes	2.9 (2.6–3.2)	3.2 (2.8–3.6)	2.6 (2.3–3.0)	
No	97.1 (96.8–97.4)	96.8 (96.4–97.2)	97.4 (97.0–97.7)	
**Stroke**				0.016
Yes	2.1 (1.9–2.4)	2.4 (2.1–2.8)	1.9 (1.6–2.2)	
No	97.9 (97.6–98.1)	97.6 (97.2–97.7)	98.1 (97.8–98.4)	
**Obesity**				0.007
Yes	35.5 (34.7–36.4)	36.7 (35.4–38.0)	34.4 (33.3–35.5)	
No	64.5 (63.6–65.3)	63.3 (62.0–64.6)	65.6 (64.5–66.7)	
**Abdominal obesity**				< 0.001
Yes	29.8 (28.9–30.7)	28.1 (26.9–29.4)	31.2 (30.3–32.5)	
No	70.2 (69.3–71.1)	71.9 (70.6–73.1)	68.8 (67.5–70.0)	
**Hypertriglyceridemia**				< 0.001
Yes	39.1 (38.2–40.0)	45.2 (43.8–46.6)	33.3 (32.2–65.5)	
No	60.9 (60.0–61.8)	54.8 (53.4–562)	66.7 (65.5–67.8)	
**Low HDL**				< 0.001
Yes	47.9 (47.0–48.9)	37.4 (36.1–38.7)	57.7 (56.5–58.9)	
No	52.1 (51.1–53.0)	62.6 (61.3–63.9)	42.3 (41.1–43.5)	

Values are shown as percentages (95% confidence intervals).

*BMI* Body Mass Index, *CI* confidence interval, *HDL* high-density lipoprotein.

*P < 0.05: for comparison between sex groups.

### The prevalence of olfactory dysfunctions

The prevalence of olfactory dysfunction is presented, stratified by age, sex, socioeconomic status, lifestyle factors (Supplementary Table S2), and CVD and its risk factors (Table 2). The overall frequency of olfactory dysfunction was 6.4% (95% confidence interval [CI] 6.0–6.9; 6.0% [95% CI 5.4–6.7] in males and 6.8% [95% CI 6.1–7.4] in females); there was no significant difference observed between the two groups. The older age group (≥ 65 years) exhibited twice the prevalence of olfactory dysfunction when compared to the middle-aged group (40–64 years). Subjects with low household income, low education levels, or that did not exercise regularly, as well as those with rhinosinusitis or rhinitis, were significantly more likely to suffer from an olfactory dysfunction; heavy drinkers, however, showed lower prevalence of olfactory dysfunction. In both sex groups, older subjects and those with low household incomes, low education levels, rhinosinusitis, or rhinitis were more likely to suffer from olfactory dysfunction, respectively. In the male group, subjects who did not exercise regularly showed a significantly high prevalence of olfactory dysfunction, whereas in the female group, subjects with a short sleep duration had a significantly high prevalence of olfactory dysfunction.

**Table 2:** Prevalence of olfactory dysfunction by history of cardiovascular disease and its risk factors among Korean adults aged 40 and over, overall and stratified by sex.

Cardiovascular disease and its risk factors	Weighted prevalence (%) of olfactory dysfunction (95% CI)		
	Total (n = 20,016)	Male (n = 8587)	Female (n = 11,429)
**Diabetes**			
Yes	8.1 (6.7–9.6)*	6.8 (5.3–8.8)	9.3 (7.4–11.7)*
No	6.2 (5.7–6.7)	5.8 (5.2–6.6)	6.4 (5.8–7.1)
P value	0.004	0.287	0.003
**Hypertension**			
Yes	7.8 (7.0–8.7)*	7.2 (6.1–8.6)*	8.3 (7.2–9.5)*
No	5.8 (5.3–6.4)	5.5 (4.8–6.4)	6.1(5.4–6.8)
P value	< 0.001	0.015	< 0.001
**CAD**			
Yes	11.6 (8.7–15.2)*	10.7 (7.1–15.9)*	12.5 (8.4–18.2)*
No	6.2 (5.7–6.7)	5.8 (5.1–6.5)	6.6 (5.9–7.2)
P value	< 0.001	0.003	0.002
**Stroke**			
Yes	9.7 (7.2–12.9)*	9.9 (6.7–14.5)*	9.4 (6.0–14.4)
No	6.3 (5.8–6.8)	5.9 (5.2–6.6)	6.7 (6.0–7.4)
P value	0.005	0.011	0.140
**Obesity**			
Yes	6.1 (5.4–6.9)	5.3 (4.4–6.5)	6.8 (5.9–7.9)
No	6.6 (6.1–7.1)	6.4 (5.7–7.3)	6.7 (6.0–7.5)
P value	0.251	0.083	0.826
**Abdominal obesity**			
Yes	7.3 (6.6–8.0*	6.6 (5.5–8.0)	7.6 (6.7–8.5)*
No	5.8 (5.3–6.4)	5.8 (5.1–6.6)	5.9 (5.2–6.7)
P value	< 0.001	0.187	0.001
**Hypertriglyceridemia**			
Yes	6.6 (5.9–7.3)	5.5 (4.6–6.4)	7.9 (6.8–9.2)*
No	6.1 (5.6–6.7)	6.1 (5.3–7.1)	6.1 (5.4–6.8)
P value	0.287	0.264	0.003
**Low HDL**			
Yes	7.2 (6.5–7.9)*	6.6 (5.5–7.9)	7.5 (6.7–8.4)*
No	5.5 (5.0–6.1)	5.4 (4.7–6.3)	5.7 (4.9–6.6)
P value	< 0.001	0.077	0.001

Values are shown as percentages (95% confidence intervals).

*CAD* coronary artery disease, *CI* confidence interval, *HDL* high-density lipoprotein.

*P < 0.05.

Subjects with diabetes, hypertension, CAD, stroke, abdominal obesity, or low HDL levels showed a significantly high prevalence of olfactory dysfunction. In both sex groups, subjects with hypertension, or CAD were likely to have olfactory dysfunction. Males with stroke had a significantly high prevalence of olfactory dysfunction, while females with diabetes, abdominal obesity, hypertriglyceridemia, or low HDL had a significantly high prevalence.

### Associations between olfactory dysfunction and CVD and its risk factors, stratified by sex

We analysed a multiple regression model for total subjects, and subsequently analysed the sex-stratified models. Multivariable adjustment for age, sex, household income, educational level, smoking status, heavy drinking, sleep duration, lack of exercise, history of rhinosinusitis and rhinitis, as well as CVD and its risk factors was done. Among the total subjects, we observed that the odds for olfactory dysfunction among those with rhinosinusitis were nine times higher than those without rhinosinusitis (odds ratio [OR] 9.33, 95% CI 7.57–11.51). Subjects with rhinitis were associated with a significantly increased OR (OR 1.64, 95% CI 1.35–1.99) compared to those without rhinitis. The older age group had a significant association with olfactory dysfunction compared to the middle-aged group (OR 1.43, 95% CI 1.17–1.74). Lower levels of education were significantly associated with olfactory dysfunction (OR: 1.35, 95% CI 1.10–1.67).

In both males and females, rhinosinusitis (in males, OR 10.23, 95% CI 7.59–13.86; in females, OR 8.61, 95% CI 6.43–11.51), rhinitis (in males, OR 1.67, 95% CI 1.25–2.21; in females, OR 1.61, 95% CI 1.25–2.12) and older age group (in males, OR 1.52, 95% CI 1.12–2.06; in females, OR: 1.32, 95% CI 1.01–1.73), were found to be significantly associated with olfactory dysfunction. In female subjects alone, with low levels of education were significantly associated with olfactory dysfunction (OR 1.40, 95% CI 1.04–1.88).

Across all subjects, a significantly increased likelihood for olfactory dysfunction was observed among those with CAD (OR 1.68, 95% CI 1.15–2.47) compared to those without. Subjects with abdominal obesity had significantly increased OR for olfactory dysfunction (OR 1.30, 95% CI 1.03–1.63) compared to those without abdominal obesity (Table 3).

**Table 3:** Multivariable-adjusted odds ratios for the association of olfactory dysfunction and cardiovascular disease and its risk factors among Korean adults aged 40 and over, overall and stratified by sex.

CVD and its risk factors	Total (n = 20,016)		Male (n = 8587)		Female (n = 11,429)	
	Adjusted OR^a^ (95% CI)	P value	Adjusted OR^b^ (95% CI)	P value	Adjusted OR‡ (95% CI)	P value
Diabetes	1.08 (0.85–1.38)	0.527	1.06 (0.74–1.51)	0.761	1.09 (0.78–1.53)	0.598
Hypertension	1.05 (0.88–1.27)	0.588	1.15 (0.86–1.53)	0.347	0.98 (0.77–1.25)	0.894
CAD	1.68 (1.15–2.47)*	**0.007**	1.81 (1.05–3.14)*	**0.034**	1.56 (0.92–2.63)	0.099
Stroke	1.33 (0.88–2.00)	0.171	1.51 (0.86–2.66)	0.153	1.11 (0.61–2.01)	0.740
Obesity	0.80 (0.64–1.01)	0.050	0.77 (0.51–1.16)	0.214	0.83 (0.64–1.06)	0.137
Abdominal obesity	1.30 (1.03–1.63)*	**0.024**	1.16 (0.78–1.72)	0.459	1.39 (1.06–1.84)*	**0.018**
Hypertriglyceridemia	1.06 (0.88–1.27)	0.568	0.96 (0.73–1.26)	0.757	1.16 (0.90–1.50)	0.246
Low HDL	1.05 (0.88–1.26)	0.578	1.07 (0.80–1.42)	0.654	1.03 (0.82–1.29)	0.795

*CAD* coronary artery disease, *HDL* high-density lipoprotein, *OR* odds ratio, *CI* confidence interval.

^a^Multivariable adjustment for age, sex, household income, educational level, smoking status, heavy drinking, sleep duration, lack of exercise, history of rhinosinusitis and rhinitis, as well as all variables shown in the table.

^b^Multivariable adjustment for age, household income, educational level, smoking status, heavy drinking, sleep duration, lack of exercise, history of rhinosinusitis and rhinitis, as well as all variables shown in the table.

*P < 0.05.

**Figure 1:**
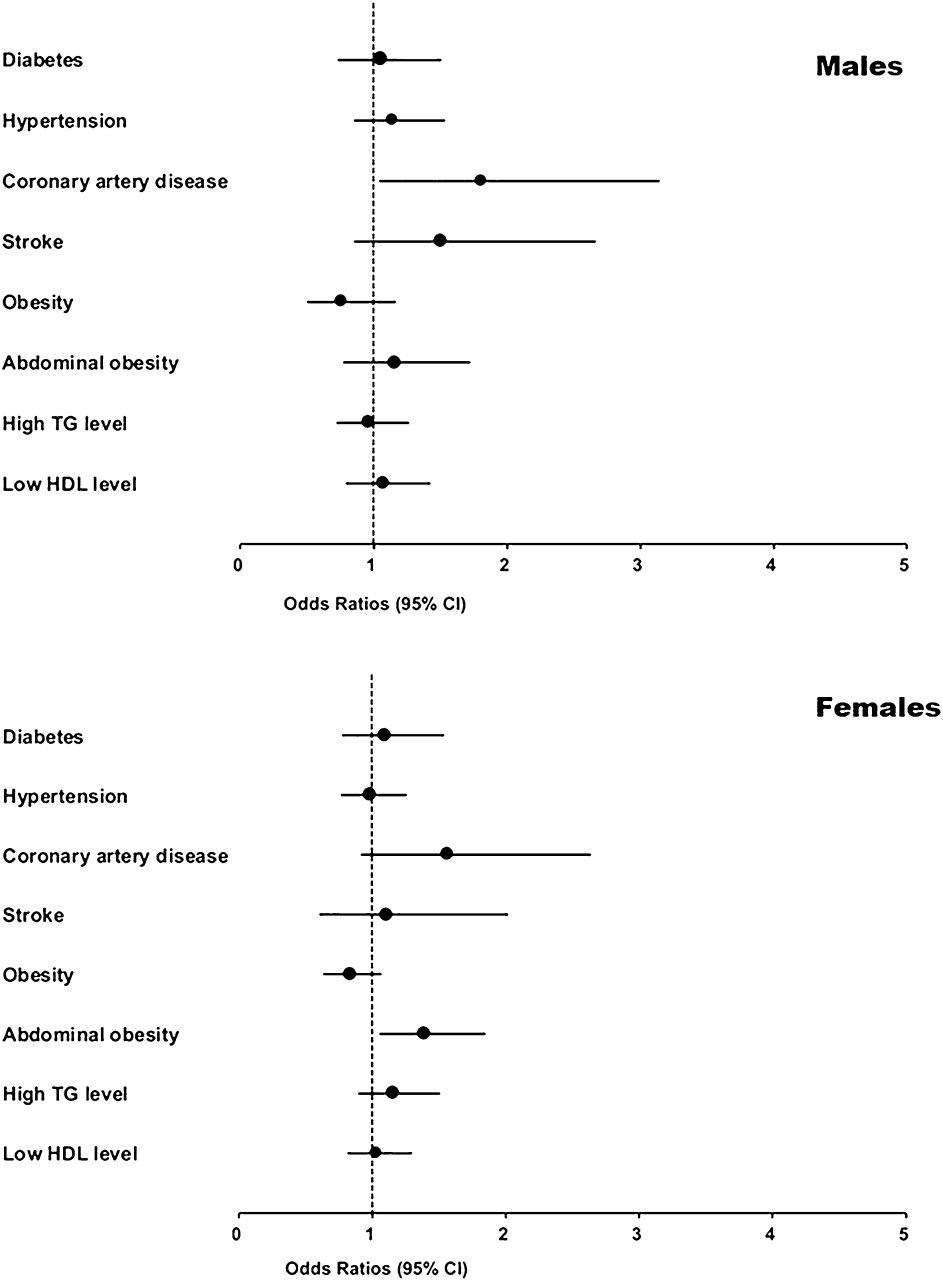
Associations of cardiovascular disease and its risk factors with olfactory dysfunction in males and females. *TG* triglyceride, *HDL* high-density lipoprotein.

Males with CAD were over 80% more likely to suffer from olfactory dysfunction than those without CAD. This ratio (OR 1.81, 95% CI 1.05–3.14) was greater than that indicated for old age (OR 1.52, 95% CI 1.12–2.06), as well as for rhinitis (OR 1.67, 95% CI 1.25–2.21). In female subjects, abdominal obesity was associated with a 39% greater likelihood for olfactory dysfunction (OR 1.39, 95% CI 1.06–1.84) than those without abdominal obesity, as well as old age (OR 1.32, 95% CI 1.01–1.73) (Fig. 1).

### The additive interaction between cardiovascular disease and its risk factors and sex in olfactory dysfunction

CAD and abdominal obesity showed a significant association with olfactory dysfunction in multivariable logistic regression analysis; hence, we calculated the interaction of these two variables with sex. Multivariable logistic regression was also used to obtain the regression coefficients and covariance matrix. Adjusted variables include age, household income, educational level, smoking status, heavy drinking, sleep duration, lack of exercise, history of rhinosinusitis and rhinitis, CVD and its risk factors except two variables (CAD or abdominal obesity, and sex) were used in interaction analysis. There was a significant interaction between abdominal obesity and female sex in this multivariable model (Table 4). The relative excess risk due to interaction (RERI) was 0.45 (95% CI 0.27–0.63), suggesting that there would be 0.45 relative excess risk due to the additive interaction between two variables. We also found that 28% of the OR of olfactory dysfunction in subjects with both variables was attributable to the additive interaction (the attributable proportion due to interaction [AP]: 0.28, 95% CI 0.1–0.47). The risk of olfactory dysfunction in females with abdominal obesity was 4.0 times as high as the sum of risks in the subjects with a single variable (abdominal obesity or female sex) (the synergy index [S]: 4.0, 95% CI 1.62–1843.93).

**Table 4:** Interaction between cardiovascular disease and its risk factors and sex for olfactory dysfunction.

Factor 1	Factor 2	OR* (95% CI)	RERI (95% CI)	AP (95% CI)	S (95% CI)
Abdominal obesity	**Sex**		0.45 (0.26–0.63)	0.28 (0.09–0.46)	4 (1.62–1843.94)
−	Male	1			
+	Male	1.19 (0.86–1.65)			
−	Female	0.96 (0.76–1.23)			
+	Female	1.60 (1.14–2.23)^†^			

*OR* odds ratio, *CI* confidence interval, *RERI* relative excess risk due to interaction, *AP* attributable proportion due to interaction, *S* synergy index.

*Multivariable adjustment for age, household income, educational level, smoking status, heavy drinking, sleep duration, lack of exercise, history of rhinosinusitis, rhinitis, diabetes, hypertension, CAD, stroke, obesity, hypertriglyceridemia, low HDL level, as well as al variables shown in the table.

^†^*P* = 0.006.

## Discussion

This study examined the prevalence of olfactory dysfunction and the associations it may have with CVD and its risk factors within the general population of middle-aged and older Koreans; the results were also stratified by sex. The interactions between CVD and its risk factors and sex were also evaluated.

We found independent associations between CAD and abdominal obesity with olfactory dysfunction. The prevalence of olfactory dysfunction was twice as high in patients with CAD, which was observed across both genders. After multivariable analysis, however, CAD was only significantly associated with olfactory dysfunction in males. Conversely, the prevalence of olfactory dysfunction according to abdominal obesity was only associated in females. Both CAD in males, and abdominal obesity in females had greater OR for olfactory dysfunction than old age, a well-known risk factor for olfactory dysfunction^1,11^. We also found abdominal obesity have additive interaction with female sex for olfactory dysfunction. This means that there will be higher association with olfactory dysfunction when abdominal obesity is present in females.

Previous studies have raised controversy surrounding the association between olfactory dysfunction and CVD^3,4,8^. A recent study using longitudinal data from older Americans reported that olfactory impairment may be a predictor for increased risk of CVD; older smokers with olfactory dysfunction over five years exhibited an increased incidence of heart attack or heart diseases. The authors suggested that olfactory dysfunction may be caused by vascular compromise at the brain level, as well as damage to the peripheral olfactory epithelium due to smoking^3^. A population-based study in older Swedish adults revealed that olfactory dysfunction was significantly associated with a history of CAD among several cardiovascular risk factors^4^; however, a population-based study among American adults aged 40 years and older showed that CVD was not significantly associated with olfactory dysfunction^8^. These inconsistent results may be a result of the varying age distributions, as well as different race, assessment methods, definitions for olfactory dysfunction, and included variables. More importantly, although considered one of the most important risk factors for olfactory dysfunction, these prior studies did not include sino-nasal disease as a variable during analysis^8,12,13^. In this study, a history of rhinosinusitis was considered the most important risk factor for olfactory dysfunction among the analysed variables. And a history of rhinitis indicated a higher likelihood for olfactory dysfunction than older age, however, it had less of an impact on olfactory dysfunction than a history of CAD within the male group.

To explain the possible mechanisms for associations of CVD and its risk factors with olfactory dysfunction, we must understand age-related olfactory neuroepithelial changes. Age-related changes include a decreased number of olfactory receptor neurons, a decrease in basal cell proliferation and replacement of olfactory neuroepithelium with respiratory epithelium^14–16^. Although the molecular basis of these changes remains unclear, several studies are investigating the basis. A decrease in basal cell proliferation with age can be explained by decreased expression of cyclin D, a transcription factor for cell proliferation, and decreased expression of growth factors such as epidermal growth factor, insulin-like growth factor-1, and neuropeptide Y signalling^16,17^ The repeated damage of olfactory receptor neurons might also be associated with decreased proliferation of basal cells after excessive basal cell divisions^18^. In addition, pro-inflammatory cytokines from senescent cells may induce the suppression of basal cell proliferation and loss of olfactory receptor neurons as aging-related physiological changes^19,20^.

Several previous studies have proposed possible mechanisms for association of CVD and its risk factors with olfactory dysfunction^12,21–23^. Firstly, vascular deficiencies related to CVD may be attributed to olfactory nerve damage^12^; a longitudinal, population-based study of older Americans by Schubert et al. reported that subclinical atherosclerosis, an important risk factor of CAD, was associated with olfactory decline and hypothesized that the atherosclerosis may be a predictor for a decline in olfactory function with age^21^. Secondly, pro-inflammatory status caused by a high-fat diet could result in olfactory dysfunction^22^; in an experimental study using mice, olfactory neurons and their axonal projections were markedly reduced after exposure to a fatty diet, and olfactory discrimination was decreased. The authors inferred that a moderately high-fat diet caused an elevation in the number of macrophage and in the degree of neuronal death, which led to reduced connections from the olfactory epithelium to the olfactory bulb^24^. Thirdly, medication for CAD may affect olfactory function; antianginal drugs and medication for cardiac diseases are known to affect olfaction^23^. However, in this study, we were unable to determine which medication had an effect on olfaction; medication details were not included with the KNHANES data.

Conversely, olfactory dysfunction might contribute to development of CVD by inducing adiposity^25–27^. Olfaction yields information about nutrients, and influence palatability and food preference, together with taste, and sense of texture^25^. The system is regulated in response to various hormones and neuromodulators such as ghrelin, orexin, neuropeptide Y, insulin, leptin, and cholecystokinin, which regulate food intake^28^; therefore, olfactory perception can influence both eating behaviour and satiety. Olfactory dysfunction may thus lead to overeating and weight gain^26,27^. Aschenbrenner et al. reported that patients with olfactory loss often altered their dietary behaviour, preferring more salty or spicy foods^29^. However, previous studies have reported inconsistent results regarding the association between olfactory dysfunction and dietary choice; Kong et al. reported that olfactory dysfunction was associated with a reduced fat intake among Korean middle-aged females^30^, whereas Schubert et al. reported that olfactory impairment was not associated with dietary choice in a large population-based study of American adults^31^.

Olfactory dysfunction has been reported to contribute to both weight loss and weight gain by influencing dietary behaviour^25^. Skrandies et al. indicated that increasing BMI was associated with a decrease in olfactory sensitivity^32^; conversely, Purdy et al. conveyed that poor olfaction was associated with lower body weight and greater weight loss in older adults^33^. A study using mice revealed that acute olfactory loss induced a metabolic response mimicking the cessation of feeding; this led to the mice being resistant to obesity by increasing thermogenesis within the fat deposits^34^. In our study, after multivariable analysis, a negative association was revealed between olfactory dysfunction and obesity, although this was not significant.

In addition to considering the association between obesity and olfactory dysfunction, some studies have investigated the relationship between abdominal obesity and olfactory dysfunction in specific^13,35^. A recent American study reported a significant association between higher waist circumference and olfactory dysfunction in middle-aged women^13^. A German study reported that abdominal obesity was inversely associated with grey matter volume, including the olfactory sulcus, although differences were not analysed according to stratification by sex^35^. In a study using an olfactometer, abdominal obesity was negatively correlated to olfactory processing speed in elderly females expressing the apolipoprotein E ε4 allele, a genetic risk factor for Alzheimer’s disease^36^. In our study, female subjects with abdominal obesity were more likely to experience olfactory dysfunction than those without.

Previous studies investigating the association between olfactory dysfunction and altered lipid profile have reported inconsistent results^5,37^. Huang et al. reported that olfactory dysfunction was associated with higher serum total cholesterol levels in Chinese adults; they suggested that higher serum total cholesterol levels in adults with olfactory dysfunction might explain the relation between olfactory dysfunction and CVD^37^. Contrary to this, Lee et al. reported that olfactory dysfunction was not associated with lipid profile in a large population-based study of Korean adults^5^. In our study, subjects with low serum HDL levels displayed a higher prevalence of olfactory dysfunction compared to those with healthy HDL levels. In the female group, subjects with hypertriglyceridemia or low serum HDL levels were more likely to experience olfactory dysfunction than those without these conditions; however, after adjusting for confounding factors, lipid profile was not found to be associated with olfactory dysfunction.

Although not investigated in this study, gene polymorphism may be associated with age-related olfactory dysfunction in human^38^. Hedner et al. investigated the effect of the brain-derived neurotrophic factor (*BDNF*) val66met polymorphism on change in olfactory function in a longitudinal population-based study. In the study, the val homozygote carriers showed larger olfactory decline compared with the met carriers in the older age cohort^38^. Dong et al. conducted the first genome-wide meta-analysis on the sense of smell among 6252 US older adults. The study revealed the microtubule-associated protein tau (*MAPT*, 17q21.31) locus may play a role in regulating olfactory dysfunction in older adults^39^. Subsequently, Dong et al. identified nine novel regions (*KLF4-ACTL7B, RAPGEF2-FSTL5, TCF4-LOC100505474, PCDH10, KIAA1751, MYO5B, MIR320B1-CD2, NR5A2-LINC00862, SALL1-C16orf97*) that were associated with olfactory function in the African-Americans and two novel loci (*RASGRP1* and *ANXA2P3*) in the European-Americans in another study of US older adults^40^.

Finally, there were inconsistent results in studies investigating the sex-linked differences in olfactory function; while some studies report that the prevalence of olfactory dysfunction in females was lower than in males, others have not observed this difference^5,8,12,31^. According to a recent meta-analysis, olfactory identification was more accurate in females than males, however, this difference was only observed in young adults and was absent in both older adults and juveniles^41^. These inconsistent findings may be a result of the differences in confounding factors, the evaluating methods for olfactory function, socio-demographic characteristics, genetic diversity of subjects, and so on. In our study, although the prevalence of olfactory dysfunction in females was higher than that in males, this was not considered significant; large differences were observed in some socio-demographic characteristics between the two genders. Females were older, with lower household incomes, lower education levels, higher prevalence of abdominal obesity, and lower HDL levels than males, whereas current smokers, heavy drinkers, and hypertriglyceridemia were more prevalent among males. These factors are known to be associated with olfactory dysfunction^1^.

Studies focusing on the association between olfactory dysfunction and CVD and its risk factors with regard to gender include one by Gallo et al. who reported an association between olfactory dysfunction and both anthropometric and cardiometabolic measures, which varied with age and gender^13^. Additionally, Hwang et al. reported that olfactory dysfunction was associated with metabolic syndrome only in women^42^. In our study, males with a history of stroke or CAD were more likely to have olfactory dysfunction than those without these conditions. Similarly, females with diabetes, CAD, a large waist circumference, hypertriglyceridemia, or low serum HDL levels were more likely to have an olfactory dysfunction than those without these conditions. After adjusting for confounders, a strong association between CAD and olfactory dysfunction was observed among the males, whereas the association between abdominal obesity and olfactory dysfunction was significant in females. Changes in gonadal hormone levels with age may contribute to the observed differences in olfactory dysfunction and cardiovascular risk factors with gender, especially in women^43–46^. Estrogen change is known to be linked to fat distribution after menopause, as well as to the prevalence of abdominal obesity which increases with age^47^.

This study had several limitations. Firstly, a cross-sectional study design cannot analyse the causal relationships between olfactory dysfunction and CVD and its risk factors. Secondly, olfactory dysfunction was assessed via an individual’s self-report, introducing the possibility of recall bias. The self-reports revealed an underestimated prevalence of olfactory dysfunction measured by objective testing; however, despite of possibility of low sensitivity, self-report measures showed high specificity and correlation to objective olfactory tests in previous studies^1,4^. Therefore, self-report assessment remains the easiest and most time- and cost-efficient method for evaluating olfactory dysfunction in large population-based studies. Thirdly, a history of head trauma and neurodegenerative disorders which may influence olfactory dysfunction^2^ were not considered confounding factors, as the KNHANES does not include the relevant data.

Nevertheless, this study had several strengths; the KNHANES is a nationwide, population-based survey with participants representative of the general Korean population aged 40 years and older. Additionally, socio-demographic characteristics, lifestyles, and comorbidities were considered, and bias was minimized by adjusting for confounding effects; in particular, a history of sino-nasal disease was included as a confounding factor during multivariable regression analysis. Lastly, this is the first study to include gender-based differences in the association between olfactory dysfunction and CVD and its risk factors with the interaction.

In conclusion, our findings suggest that CAD may be a predictor for olfactory dysfunction in males, whereas a large waist circumference may be a predictor for the disease in females, among middle-aged and older adults. Moreover, this study further showed an additive interaction between abdominal obesity and female sex, highlighting that females with abdominal obesity are significantly associated with olfactory dysfunction Therefore, health service providers should consider the risk of olfactory dysfunction possibly afflicting males with CAD and females with abdominal obesity. Conversely, clinicians and policymakers should pay attention to the likelihood and prevention of cardiovascular disease in individuals with olfactory dysfunction. As both olfactory dysfunction and CVD could potentially result in critical health outcomes within the aging global population, further prospective studies are required to clearly establish the link between CVD and olfactory dysfunction, and the related underlying mechanisms.

## Methods

### Study population

The Korean National Health and Nutrition Examination Survey (KNHANES) is a nationwide, population-based, cross-sectional health examination survey, conducted by the Division of Chronic Disease Surveillance under the auspices of the Korea Centers for Disease Control and Prevention (KCDC). The KNHANES is conducted to evaluate the health and nutritional status of non-institutionalized Korean citizens. The health interview and health examination are performed by trained medical staff and interviewers in mobile examination centers. Peripheral blood samples were collected after fasting for more than eight hours, and then were immediately processed and transported to a central laboratory (Neodin Medical Institute, Seoul, South Korea). All blood samples were analysed within 24 h after collection^48^.

We used data collected during the 2008–2012 KNHANES. Of the 23,612 subjects (≥ 40 years of age) who participated in the survey, 20,016 completed the clinical examination, the health questionnaire, and a questionnaire of olfactory symptoms (Supplementary Fig. S1). All subjects provided written informed consent for use of their data prior to survey commencement. The study was approved by the Institutional Review Board of the KCDC (numbers 2008–04EXP-01-C, 2009–01CON-03-2C, 2010–02CON-21-C, 2011–02CON-06-C and 2012–01EXP-01-2C) and performed in accordance with relevant guidelines/regulations.

### Socio-demographic characteristics and lifestyle factors

The health interview evaluated socioeconomic characteristics (household income and educational level), lifestyle (smoking, alcohol drinking, sleeping duration, and exercise status), and comorbidities using self-report questionnaire. Smoking history was categorized as current smoker or non-smoker. Alcohol use was based on the amount of alcohol consumed per day during the month before the interview. Males who drank seven or more glasses of alcohol more than twice per week and females who drank five or more glasses more than twice per week were categorized as heavy drinkers. Regular exercise was defined as moderate physical activity performed for at least 30 min at a time at least five times a week. Height, weight, and waist circumference were measured by trained medical professionals^48^.The body mass index (BMI) was calculated as body weight (kg) divided by height in meters squared (m^2^)^49^.

### Definitions of olfactory dysfunction

All subjects were asked about their history of anosmia or hyposmia as follows: “Have you had problems with your sense of smell for more than 3 months during the past 12 months?” Subjects who answered “yes” were considered to have olfactory dysfunction.

### CVD and its risk factors

All subjects were asked about their history of CVD and its risk factors. The lifetime prevalence of CVD such as coronary artery disease (CAD) and stroke was assessed by a physician. CVD risk factors such as diabetes (current use of insulin or oral medication to treat elevated glucose, fasting blood glucose ≥ 126 mg/dL or history of diabetes), hypertension (systolic blood pressure and/or diastolic blood pressure ≥ 140/90 mmHg, self-reported current use of antihypertensive medication or history of hypertension), hypertriglyceridemia (fasting serum triglyceride ≥ 200 mg/dL or current relevant medication regimen), low serum HDL level (HDL cholesterol < 40 mg/dL or current relevant medication)^50^, obesity (BMI ≥ 25 kg/m^2^)^49^, abdominal obesity (waist circumference ≥ 90 cm in male and waist circumference ≥ 85 cm in female)^49^ were included to identify possible influences on olfactory dysfunction.

### Statistical analysis

We performed statistical analysed using the complex sample analysis procedure of PASW software (ver. 18.0; SPSS Inc., Chicago, IL, USA) to reflect the complex sampling design and sampling weights of the KNHANES, and to provide nationally representative prevalence estimates. Results are presented as percentages for categorical variables and as estimated means ± standard deviation for continuous variables. To compare categorical and continuous variables, the chi-squared test and a general linear model, respectively, were used. The multivariable analysis employed logistic regression to explore the associations of olfactory dysfunction with CVD and its risk factors. Multivariable adjustment was for age, sex, household income, educational level, smoking status, alcohol consumption, sleep duration, exercise status, and the history of rhinosinusitis and rhinitis, because these variables have previously been associated with olfactory dysfunction^12,51,52^. We calculated the ORs for olfactory dysfunction Again, after stratification by sex, we calculated OR for olfactory dysfunction with multivariable adjustment for all variables above except sex. To examine the interaction between CVD and its risk factors with sex for olfactory dysfunction, multivariable logistic regression was used to obtain the regression coefficients and covariance matrix^53^. Adjusted variables include age, household income, educational level, smoking status, heavy drinking, sleep duration, lack of exercise, history of rhinosinusitis and rhinitis, CVD and its risk factors except two variables (one of CVD and its risk factors which was found to be significant associated with olfactory dysfunction and sex) to be used in interaction analysis. We use Microsoft Excel sheet to calculate three measures: the relative excess risk due to interaction (RERI); the attributable proportion due to interaction (AP); and the synergy index (S)^54,55^.

## Supplementary Information

Supplementary Information.

## Data Availability

The authors used 2008–2012 KNHANES data. All files are available from the KNHANES webpage (URL:https://knhanes.cdc.go.kr/knhanes/eng/index.do;jsessionid=U1mFHXN2dzfKNNVOvQKB0nmYaQqn05mBwm108CngA0EMjVKaMeFyOGtRg1Eelh7d.KCDCWAS01_servlet_PUB1). All other relevant data are within the paper and its Supporting Information files.
