# Supplementary material for: The association between olfactory dysfunction and cardiovascular disease and its risk factors in middle-aged and older adults
Source: Sci Rep. 2021 Jan 13;11:1248. doi: 10.1038/s41598-020-80943-5 (PMC7806612; doi:10.1038/s41598-020-80943-5)
Supplement: Supplementary file 1 — Supplementary Information. [file 41598_2020_80943_MOESM1_ESM.pdf]

The association between olfactory dysfunction and cardiovascular disease and its risk factors in middle-aged and older adults

Daeyoung Roh[1], M.D., Ph.D., Dong-Hee Lee[2], M.D., Ph.D., Soo Whan Kim[2], M.D., Ph.D., Sung Won Kim[2], M.D., Ph.D., Byung-Guk Kim[2], M.D., Ph.D., Do Hyun Kim[2], M.D., Ph.D. and Ji-Hyeon Shin[2,*] , M.D., Ph.D.

[1] Mind-neuromodulation Laboratory and Department of Psychiatry, Chuncheon Sacred Heart Hospital, Hallym University College of Medicine, Chuncheon-si, Gangwon-do, Republic of Korea

[2] Department of Otolaryngology-Head and Neck Surgery, College of Medicine, The Catholic University of Korea, Seoul, Republic of Korea

Corresponding Author:

Address correspondence and reprint requests to Ji-Hyeon Shin, M.D., Ph.D.

Department of Otolaryngology-Head and Neck Surgery, College of Medicine, The Catholic University of Korea, 222 Banpo-daero, Seocho-gu, Seoul, 06591, Republic of Korea.

E-mail: tachyon0217@gmail.com

Tel: 82-31-820-3892

Fax: 82-31-847-0038

Supplementary Figure S1. Flow chart showing the selection of study participants.

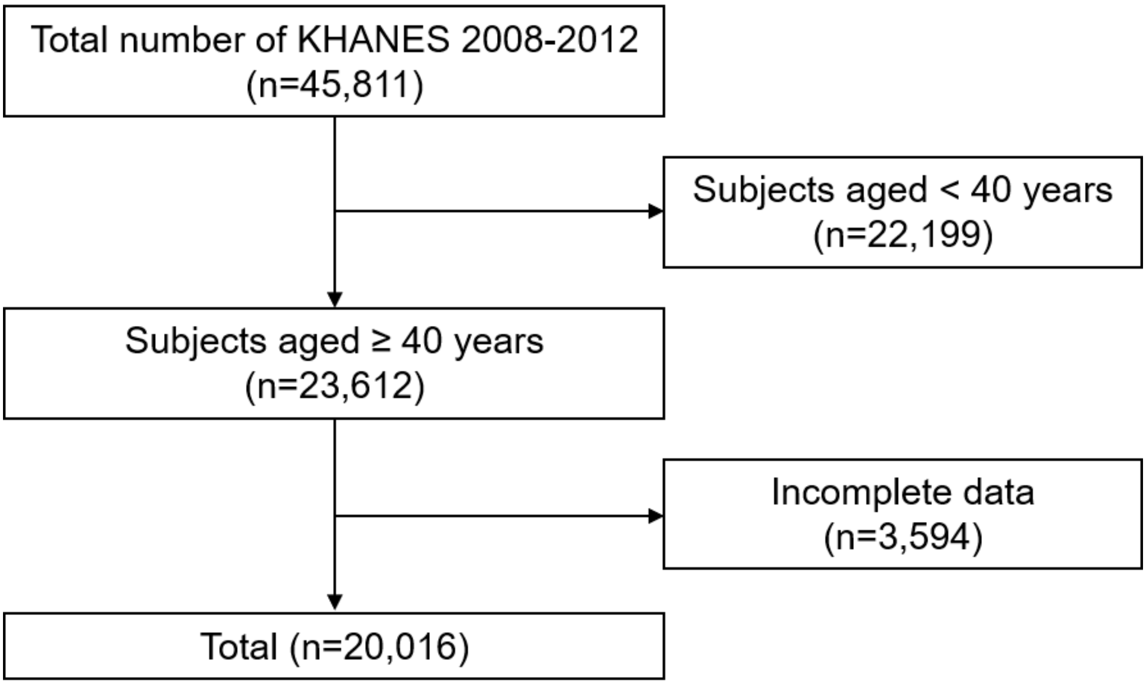

Supplementary Table S1. Sociodemographic and clinical characteristics among Korean adults aged 40 and over, overall and stratified by sex

	Weighted prevalence % (95% CI)			P value for comparison between sex groups
**Characteristics**	**Total (n=20016)**	**Male (n=8587)**	**Female (n=11429)**	
**Age**				<0.001
Mean ± SD	55.8 ± 0.1	54.8 ± 0.2	56.6 ± 0.2	
40-64 years	76.0 (75.2-76.7)	79.4 (78.4-80.4)	72.9 (71.8-73.9)	
≥65 years	24.0 (23.3-24.8)	20.6 (19.6-21.6)	27.1 (26.1-28.2)	
**Household income**				<0.001
≤ Lower quartile	21.7 (20.9-22.5)	18.1 (17.1-19.2)	24.9 (23.7-26.2)	
> Lower quartile	78.3 (77.5-79.1)	81.9 (80.8-82.9)	75.1 (73.8-76.3)	
**Education level**				<0.001
≤ Middle school	47.8 (46.6-48.9)	37.5 (35.9-39.1)	57.2 (55.7-58.8)	
> Middle school	52.2 (51.1-53.4)	62.5 (60.9-64.1)	42.8 (41.2-44.3)	
**Current smoking**				<0.001
Yes	29.8 (28.9-30.7)	55.4 (54.0-56.7)	6.3 (5.8-6.9)	
No	70.2 (69.3-71.1)	44.6 (43.3-46.0)	93.7 (93.1-94.2)	
**Heavy drinking**				<0.001
Yes	12.0 (11.4-12.7)	21.8 (20.7-23.0)	3.1 (2.7-3.5)	
No	88.0 (87.3-88.6)	78.2 (77.0-79.3)	96.9 (96.5-97.3)	
**Sleep duration**				<0.001
≤6 hours	44.0 (43.1-44.9)	41.4 (40.0-42.7)	46.4 (45.2-47.6)	
>6 hours	56.0 (55.1-56.9)	58.6 (57.3-60.0)	53.6 (52.4-54.8)	
**Lack of exercise**				<0.001
Yes	72.9 (71.8-73.9)	70.0 (68.4-71.5)	75.3 (73.9-76.6)	
No	27.1 (26.1-28.2)	30.0 (28.5-31.6)	24.7 (23.4-26.1)	
**Rhinosinusitis**				<0.001

Yes	6.3 (5.9-6.8)	7.4 (6.7-8.2)	5.3 (4.7-5.8)	
No	93.7 (93.2-94.1)	92.6 (91.8-93.3)	94.7 (94.2-95.3)	
**Rhinitis**				0.341
Yes	21.6 (20.8-22.4)	22.0 (20.7-23.3)	21.2 (20.1-22.3)	
No	78.4 (77.6-79.2)	78.0 (76.7-79.3)	78.8 (77.7-79.9)	

Values are shown as percentages (95% confidence intervals)

*p <.05: for comparison between sex groups.

CI: confidence interval; SD: standard deviation.

Supplementary Table S2. Prevalence of olfactory dysfunction by sociodemographic and clinical characteristics among Korean adults aged 40 and over, overall and stratified by sex

	Weighted prevalence % (95% CI)		
Characteristics	Total (n=20016)	Male (n=8587)	Female (n=11429)
**Age**			
40-64 years	5.2 (4.8-5.8)*	4.9 (4.3-5.7)*	5.5 (4.9-6.3)*
≥65 years	10.1 (9.2-11.1)	10.1 (8.7-11.8)	10.0 (8.9-11.3)
P value	<0.001	<0.001	<0.001
**Household income**			
≤ Lower quartile	9.1 (8.1-10.1)*	8.8 (7.2-10.6)*	9.3 (8.1-10.6)*
> Lower quartile	5.6 (5.1-6.1)	5.4 (4.7-6.2)	5.7 (5.1-6.5)
P value	<0.001	<0.001	<0.001
**Education level**			
≤ Middle school	8.1 (7.4-8.8)*	7.8 (6.7-9.0)*	8.3 (7.5-9.2)*
> Middle school	4.7 (4.2-5.3)	4.8 (4.1-5.7)	4.6 (3.8-5.5)
P value	<0.001	<0.001	<0.001
**Current smoking**			
Yes	6.0 (5.3-6.9)	5.8 (5.0-6.7)	8.0 (5.8-10.9)
No	6.4 (5.9-7.0)	6.1 (5.2-7.1)	6.6 (5.9-7.3)
P value	0.433	0.664	0.264
**Heavy drinking**			
Yes	5.1 (4.0-6.3)*	5.1 (4.0-6.6)	4.6 (2.6-8.0)
No	6.5 (6.0-7.0)	6.1 (5.4-7.0)	6.7 (6.1-7.4)
P value	0.035	0.177	0.179
**Sleep duration**			
≤6 hours	6.6 (6.0-7.3)	5.9 (5.1-6.9)	7.1 (6.3-8.1)*
>6 hours	6.1 (5.6-6.8)	5.9 (5.1-6.8)	6.4 (5.6-7.2)

P value	0.069	0.948	0.155
**Lack of exercise**			
Yes	6.7 (6.2-7.4)*	6.6 (5.8-7.6)*	6.8 (6.1-7.7)
No	5.6 (4.8-6.5)	4.9 (3.8-6.2)	6.4 (5.3-7.7)
P value	0.033	0.028	0.519
**Rhinosinusitis**			
Yes	35.2 (31.9-38.6)*	33.4 (28.8-38.2)*	37.5 (32.8-42.4)*
No	4.5 (4.1-4.9)	3.8 (3.3-4.4)	5.1 (4.5-5.7)
P value	<0.001	<0.001	<0.001
**Rhinitis**			
Yes	11.6 (10.4-12.9)*	11.3 (9.6-13.2) *	11.9 (10.4-13.7)*
No	5.0 (4.5-5.5)	4.5 (3.9-5.2)	5.4 (4.8-6.1)
P value	<0.001	<0.001	<0.001

Values are shown as percentages (95% confidence intervals)

*p <.05: for comparison between sex groups.

CI: confidence interval
